# Interplay between Chiro-Optical and Spin Transport Properties in Chiral CdSe Quantum Dots

**DOI:** 10.1021/acsnano.5c12765

**Published:** 2025-09-22

**Authors:** Elizabeth Shiby, Rui Sun, Brian P. Bloom, Joseph A. Albro, Dali Sun, David H. Waldeck

**Affiliations:** † Department of Chemistry, 6614 University of Pittsburgh, Pittsburgh, Pennsylvania 15260, United States; ‡ Department of Physics, 6798 North Carolina State University, Raleigh, North Carolina 27695, United States

**Keywords:** chiral-induced spin-selectivity, chiral quantum dots, spin-polarized charge current, pure spin current, chirality-induced unconventional spin−orbit coupling

## Abstract

The chiral-induced spin selectivity (CISS) effect offers compelling approaches for manipulating spin-dependent processes in both chemistry and physics, yet the physical mechanism(s) underpinning CISS are still debated. Here we present a study of structure–property relationships in chiral quantum dot assemblies to identify attributes of CISS-based phenomena. Our results show that the circular dichroism (CD) strength of chiral CdSe quantum dots’ primary exciton transition correlates with two CISS properties, the propensity to transmit pure spin currents and to produce spin-polarized charge currents. While the spin-polarized charge current reverses its sign with a change in the polarity of the CD signal, the pure spin current transport does not. The trends in both transport types can be rationalized in terms of chirality-induced splitting of spin sub-bands, which are modulated by chiral symmetry breaking under charge transport through the CdSe quantum dots.

## Graphical abstract

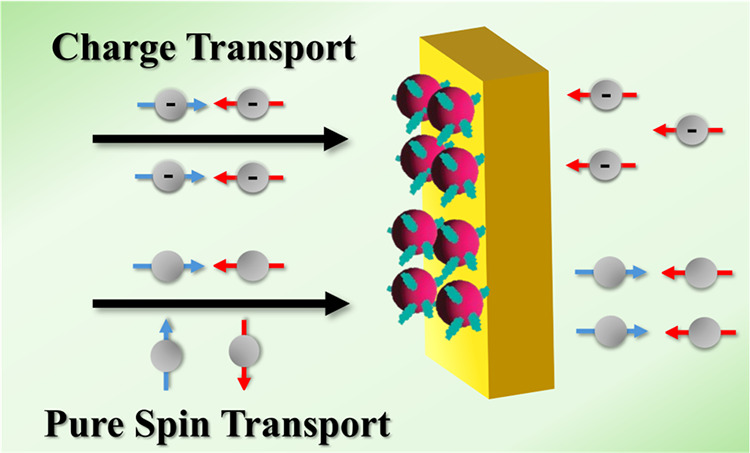

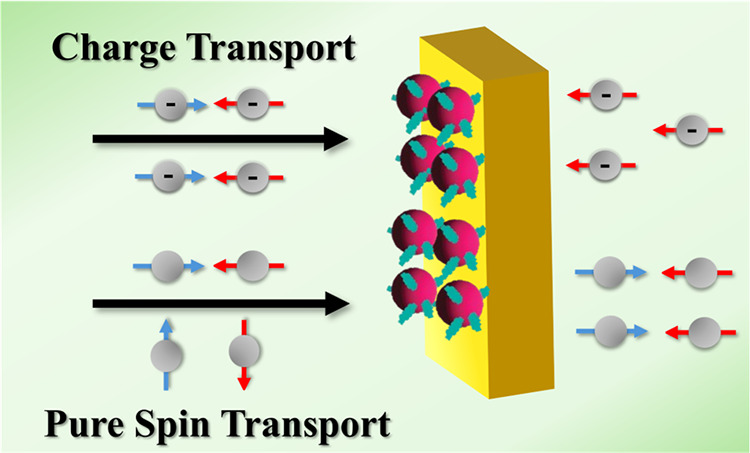

## Introduction

The CISS effect refers to the fact that electron spin transport through chiral materials is enantiospecific; that is, when electrons pass through a chiral material one spin is preferentially transmitted over the other and which spin is preferred depends on the enantiomeric form of the material.^1–3^ This phenomenon has been observed in a diverse set of molecules, polymers, and semiconductors including hybrid perovskites, semiconductor nanoparticles, metal oxides, among others.^4–9^ The CISS effect enables new paradigms for chiral-(opto)­spintronics, including energy-efficient photodetectors for circularly polarized light and spin-light-emitting diodes (spin-LEDs), and for realizing novel room temperature spintronic devices, including spin valves, memristors, and nonvolatile memory devices that do not require a permanent magnet.^10–21^ While considerable progress in understanding the CISS phenomenon has been made in recent years, a quantitatively accurate *ab initio* description remains elusive,^1,22–25^ in part, due to a lack of structure–property relationships for chiral materials that identify necessary features of a comprehensive CISS theory.

Among all of the CISS-active materials candidates, cadmium selenide (CdSe) quantum dots (QDs) provide a useful model system for exploring CISS because of the well-established chemical synthetic routes for imparting chirality^26–29^ and their proven spin filtering attributes.^30–34^ Spin-dependent electron transport through CdSe QDs was first shown using magnetic conductive atomic force microscopy (mcAFM) and magnetoresistance devices.^30^ Those findings reported spin filtering of charge currents through chiral CdSe QD films with an asymmetry of up to 30%. In subsequent work, donor-bridge-acceptor assemblies of QDs -featuring achiral donors tethered to chiral CdSe acceptors - exhibited chirality dependent photoinduced electron transfer rates. These rates correlated with the strength of the circular dichroism (CD) signal of the acceptors’ primary excitonic transition and displayed asymmetries of up to 88%.^31^ While precedent exists for the efficient spin-filtering effect in charge transport, the pure spin transport in chiral QDs, which can be governed by structural chirality,^35^ has not been probed.

The ability to direct pure spin currents in a material offers new design possibilities for next generation spintronic applications. We recently examined pure spin current transport in chiral cobalt oxide films and showed that the pure spin current’s transmissivity and the structural chiral axis align preferentially with the surface normal.^35^ As reported in studies of many other chiral materials and molecular assemblies,^4–9,36–38^ mc-AFM measurements of chiral cobalt oxide films showed a spin-dependent charge current whose current dissymmetry changes its sign with the film’s handedness (enantiomorph), but does not change with voltage bias; *i.e*., it changes from about 25% for L-cobalt oxide to −25% for D-cobalt oxide. In contrast, the pure spin current absorption was independent of the film’s enantiomorph and the sign of the external magnetic field.^35^ The spin current showed a strong dependence on spin polarization angle θ_*M*_ with respect to the surface normal; *i.e*., the spin current absorption was proportional to λ·cos^2^ θ_*M*_, where λ is a dissymmetry factor that represents the difference in pure spin current absorption when spin injection is perpendicular or parallel to the surface normal (*vide infra*). That is, the spin current absorption in the chiral cobalt oxide film exhibited a maximum (minimum) value when the direction of the spin polarization was parallel (perpendicular) to the film’s surface normal, by about 30 times. In related work, researchers have reported anisotropic absorption of spin currents for chiral molecule films^39,40^ and chirality-induced pure spin injection with a longitudinal spin-to-charge conversion in chiral polymers (*i.e*., inverse CISS effect).^41^ The importance of pure spin current transport for low-energy spintronic applications, the observation of strong asymmetries in pure spin transport, and their contrast with asymmetries observed in spin-filtered charge currents, highlights the need for a unified and universal physical framework to understand the interplay among structural chirality, charge transport, and pure spin current transport.

In this work we investigate how chirality can be used to modulate spin-dependent charge current and pure spin current transport; see Scheme 1. A series of chiral CdSe QDs were synthesized and the anisotropy of their circularly polarized light absorption, *i.e*. circular dichroism response, was used to quantify the chirality (pink). By systematically tailoring the CdSe QDs chiroptical response the effect of chirality on spin polarized charge currents (blue) and pure spin current transport (green) was investigated. Our findings reveal that the spin filtering preference for spin-polarized charge current strongly correlates with the CD strength of the QDs’ first exciton transition and reverses sign with their handedness (l- vs d-). Conversely, pure spin current absorption correlates with the magnitude of the CD strength, but its sign remains independent of the QDs’ handedness. These findings highlight the rich interplay between the degree of a material’s chirality and the transmission of pure spin and spin-polarized charge currents, deepening the physical understanding of the CISS effect in chiral materials.

**Scheme 1:**
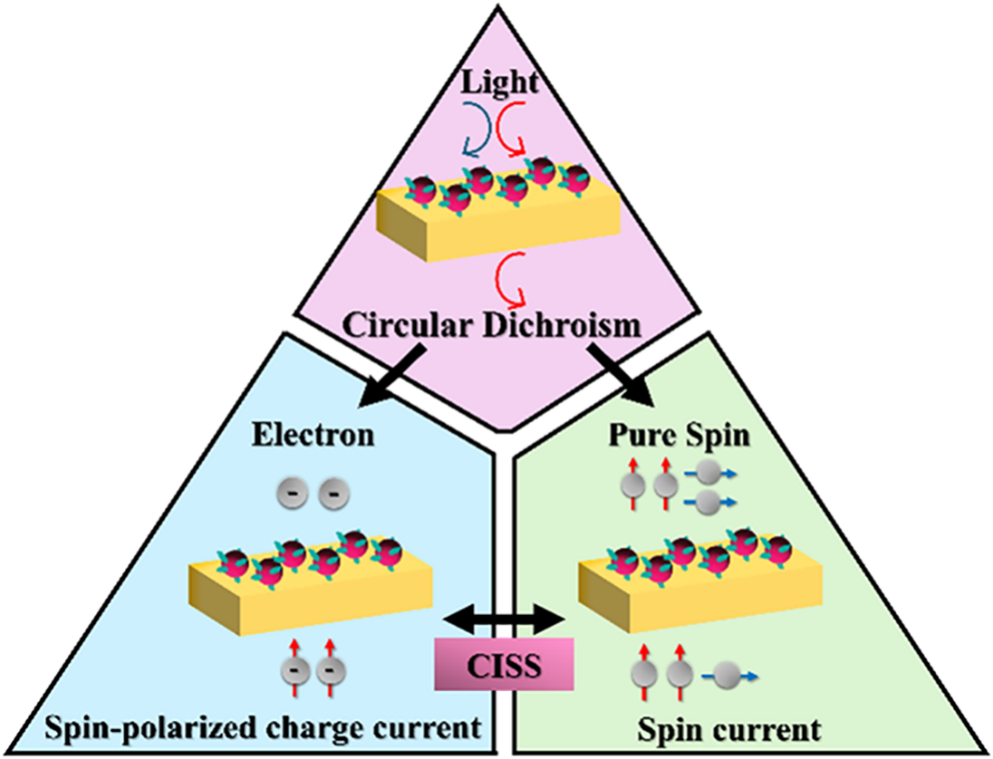
Schematic Illustration of the Relationship between Chirality and Transport through Chiral CdSe Quantum Dot Films^a^

a The chiroptical properties of CdSe QDs (red spheres) were measured using circular dichroism spectroscopy and the magnitude of anisotropy in absorption of circularly polarized light was correlated with their propensity to affect pure spin current absorption (green) and transmit spin polarized charge currents (blue).

## Results

Colloidal CdSe QDs were synthesized following previously published protocols,^42^ and the ODPA native ligands were exchanged with l-cysteine (d-cysteine) to create chiral l-cys CdSe (d-cys CdSe) QDs and with mercaptopropionic acid (MPA) to create achiral QDs (MPA CdSe), which were used as an achiral control sample (see the Methods section for more details).^43^ Figure S1 shows representative absorbance (a) and circular dichroism (CD) spectra (b) of the resulting l-cys CdSe (black), d-cys CdSe (red), and MPA CdSe (pink) QDs dispersed in water. The first exciton peak at 512 nm is consistent with 2.46 nm diameter QDs. The mirror image bisignate feature at the first excitonic transition for the L- and d-cys CdSe QDs in the CD spectra confirms that the ligands imprint chirality onto the electronic states of the QD and are consistent with that shown in other works.^43,44^ The achiral control sample, MPA CdSe QDs display no significant CD signal.

### Spin-Dependent Charge Transport Measurements

To perform spin dependent charge transport in these QD films, a self-assembled monolayer (SAM) was formed on a gold-coated silica substrate using 8-amino-1-octanethiol, and then nanomolar concentrations of CdSe QDs were dropcast to form an assembly on the SAM (see Figures 1a and S2). The mcAFM measurements were then performed on the assembly using a magnetized Co–Cr tip, which was magnetized either North or South,^45^ and the asymmetry in transport of the spin-polarized charge currents through the material was studied (see Figure S3 and Section S1 for a schematic diagram of the measurement setup and its working mechanism). To ensure that the data does not suffer from experimental drift and tip degradation, three sets of *I–V* curves were collected by alternating the magnetization of the tip, with each set consisting of an average *I–V* response of at least 30 different locations on the QD assemblies. A data set was considered to be valid only if the first and third sets of curves (measured using the same magnetizations) were within the 95% confidence interval of each other.

**Figure 1:**
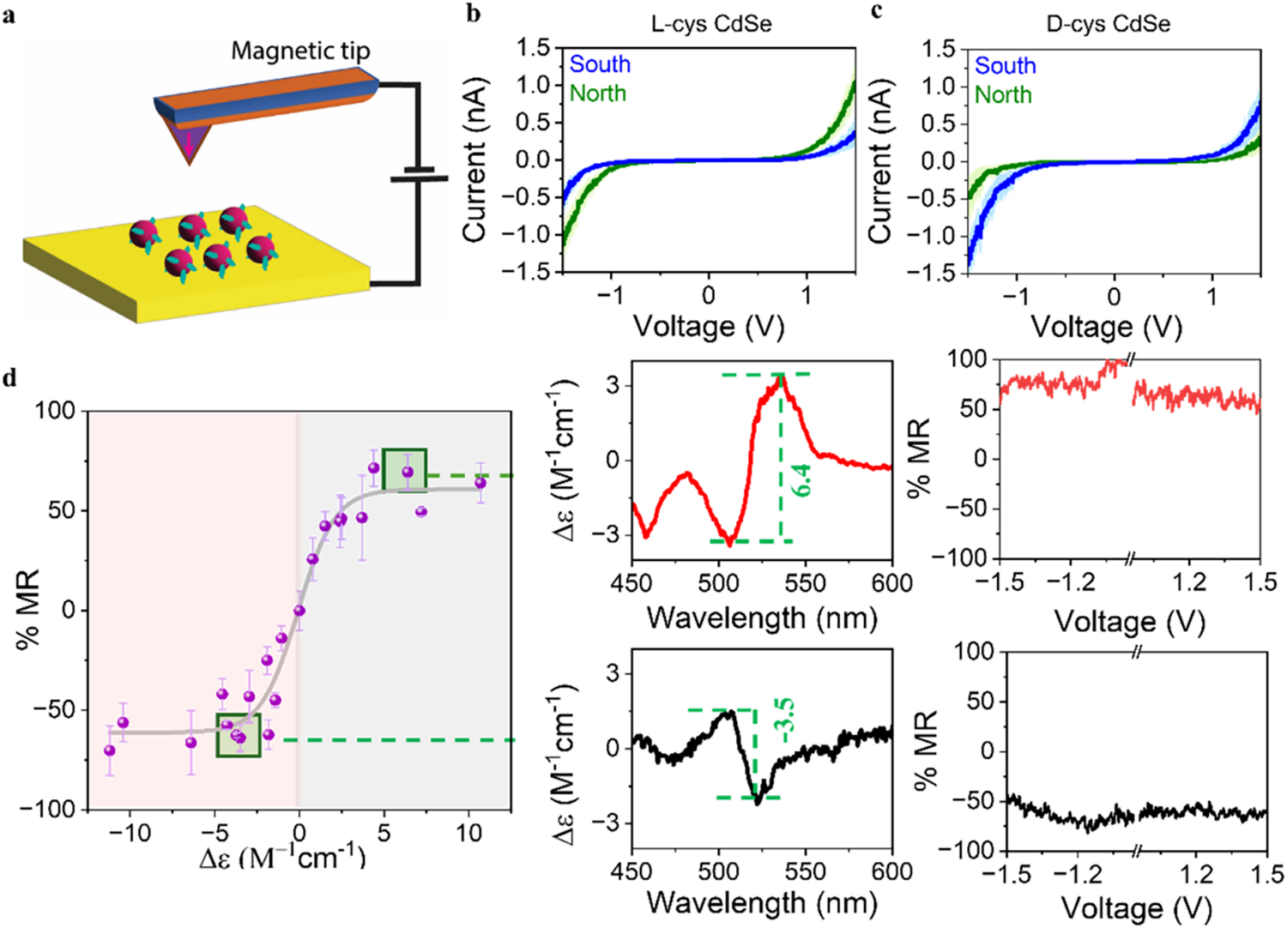
Spin-polarized charge current measurements. (a) Schematic diagram of an mc-AFM setup with a magnetic tip. mcAFM measurements for l-cys CdSe QDs (b) and d-cys CdSe QDs (c). The solid line corresponds to the average current response and the shaded region represents the 95% confidence interval of the data. (d) %MR versus the molar CD strength in chiral CdSe QDs (left). The gray line represents a sigmoidal fit to the data, and the error bars represent the standard deviation from the average value. The graphs on the right show representative CD spectra, in units of molar extinction, and %MR data for a batch of d-cys CdSe (top) and l-cys CdSe QDs (bottom), respectively.

Figure 1b,c show the statistically averaged *I–V* curves for chiral l-cys and d-cys CdSe QDs films that were measured using a tip magnetized North (green) and South (blue) with a permanent magnet, respectively. Note that the shaded region represents the 95% confidence interval of the data. For l-cys CdSe QDs, a higher (lower) charge current was observed when the tip was magnetized with the North (South) pole of the magnet. In contrast, for the d-cys CdSe QDs, the opposite response was observed, i.e., a higher current for the tip magnetized South and a lower current for the tip magnetized North. As for the control MPA-CdSe QD sample, no appreciable differences in the current with magnetization was observed. The behavior with the magnetization direction of the tip coincides with that observed in previous CISS-active spin filtering experiments of chiral CdSe QDs; i.e., d-cys CdSe preferentially transmits spins antiparallel to the momentum of the electron, whereas l-cys CdSe preferentially transmits spins parallel to the momentum of the electron.^8,9^ The resulting asymmetry in charge current with magnetization direction can be converted to a percent magnetoresistance, %MR, using eq 1;

1 $$\%\mathrm{MR}=1.3\times \frac{I_{S}-I_{N}}{I_{S}+I_{N}}\times 100\%$$

where *I*_S_ and *I*_N_ correspond to the average current measured when the tip is magnetized using the South and North poles of the magnet, respectively. The numerical factor 1.3 is a correction factor to account for vacancies in the assembly that detract from the magnetoresistance (see Section S2 for an additional discussion of this point).

To study the relationship between the CD strength and the %MR of the CdSe QDs, a 3-fold strategy was employed to create QDs with varying degrees of chirality: (i) the size of the particles was varied, since this has been shown to affect the CD strength;^44^ (ii) the enantiopurity of the ligand shell was altered systematically by adjusting the enantiomeric ratio during ligand exchange;^31^ and (iii) the concentration of ligand used during the ligand exchange was varied, which can cause changes in the binding mode of the ligand to the CdSe and hence the CD intensity.^46^ In each case, the CD spectra of the QDs were measured, and the CD strength was determined by taking the difference in Δε at the low energy and high energy lobes of the bisignate feature associated with the first excitonic transition of the QDs. Figure 1d (left) plots the %MR data for 23 separately prepared QD batches vs the CD intensity in units of molar extinction. The data on the right show plots of %MR versus applied voltage and CD spectra for a representative d-cys CdSe (top) and l-cys CdSe (bottom) data point. For QDs with CD strength over a range of approximately ± 2.5 M^–1^ cm^–1^, the %MR increases nearly linearly from −50% at −2.5 M^–1^ cm^–1^ to 50% at 2.5 M^–1^ cm^–1^. Outside of this range the %MR saturates with a maximum at approximately 60% for d-cys CdSe QDs and −60% for l-cys CdSe QDs. Despite the highly variable manner in which the CD strength of the QDs was attained (different particle sizes, ligand coverages, and ligand shell enantiopurity), the data appear to follow a universal curve and can be fit by a sigmoid function (gray). The quality of the fits and the consistent trend in the data demonstrate that the CD strength is a reasonable metric for predicting the %MR values.

### Spin Pumping Measurements

Figure 2a shows a schematic diagram of the ferromagnetic resonance (FMR) experiment. In this experiment, microwaves drive the precession of magnetization in a 15 nm Ni_81_Fe_19_(NiFe) layer under the resonance condition and the resonance is damped by injecting a pure spin current, *J*_*s*_, to the adjacent chiral layer indicated by the damping factor extracted from the FMR line width. Using a vector network analyzer (VNA-FMR), we conducted broadband FMR measurements to obtain the damping factor for 15 nm thick films of MPA-CdSe, l-cys CdSe, and d-cys CdSe QDs. The data were then fit using the Landau-Lifschitz-Gilbert equation, which is known to describe the magnetization dynamics of permalloy (Ni_81_Fe_19_), to obtain the damping factor, α (see the Supporting Information (SI) for the details). The damping factor of the permalloy’s magnetization is dominated by pure spin current transport into the CdSe QD film. Figure 2b shows examples of the FMR spectra, 10 GHz microwave absorption versus the applied magnetic field *H*, for the NiFe/MPA CdSe (pink), NiFe/l-cys CdSe (black), and NiFe/d-cys CdSe (red) films with the external magnetic field oriented along the in-plane direction, respectively. The NiFe/l-cys CdSe and NiFe/d-cys CdSe exhibit broader line widths than that of MPA CdSe QD films, indicating a higher damping factor for the chiral films, and imply more efficient pure spin current injection into the chiral CdSe QD films than into the analogous achiral films.

**Figure 2:**
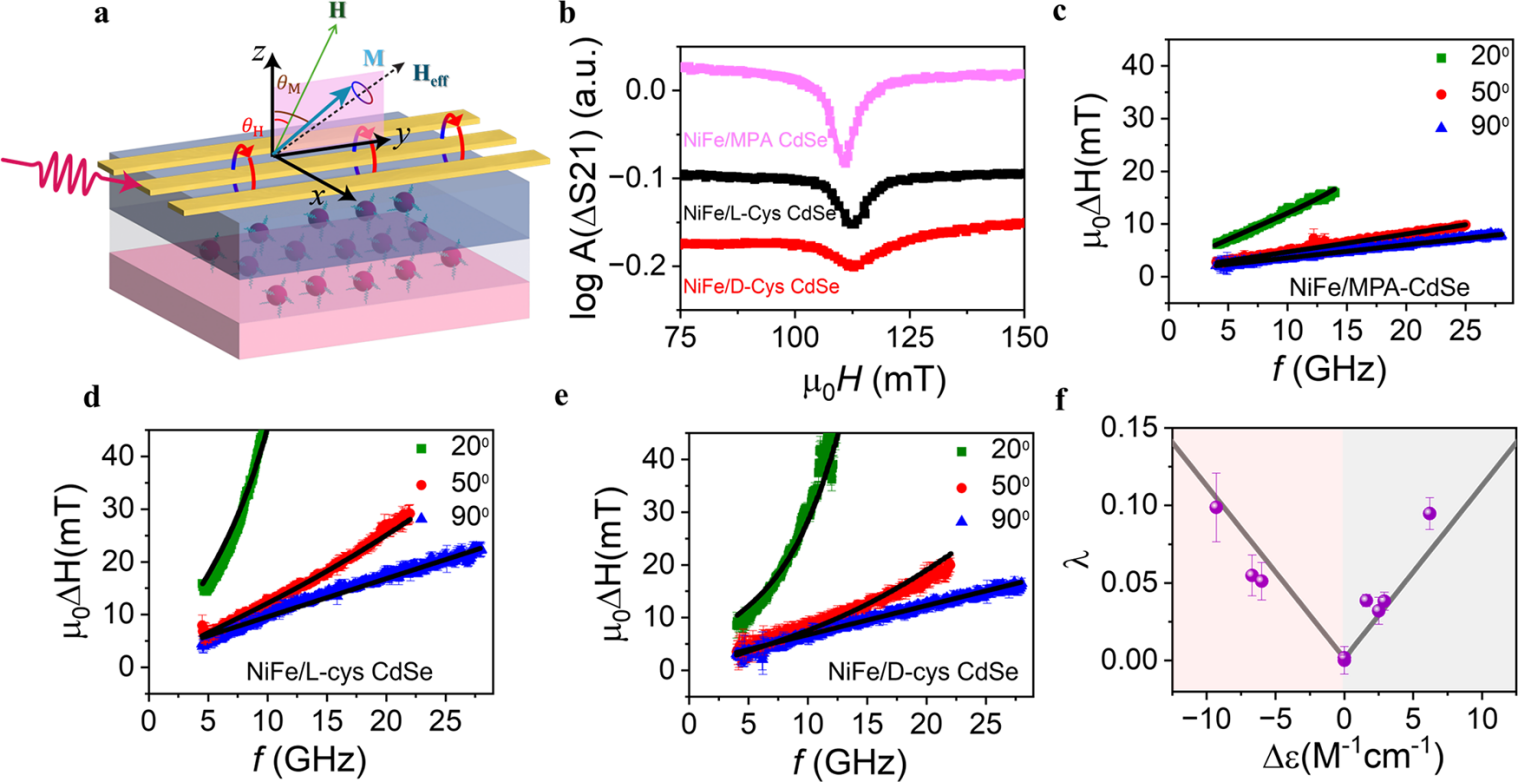
Pure spin current measurements. (a) Schematic diagram of the FMR setup. The pure spin current *J*_*s*_ is injected from the NiFe layer into the CdSe QD film. (b) Representative examples of FMR spectra measured at 10 GHz, θ_*H*_ = 90°, 300 K for the NiFe/MPA CdSe, NiFe/d-cys CdSe,and NiFe/l-cys CdSe. (c–e) dependence of line width Δ*H* on frequency *f* at different field angles, θ*_H_* = 90, 50, and 20°, for NiFe/MPA CdSe (c), NiFe/l-cys CdSe (d), and NiFe/d-cys CdSe (e). The solid lines are a fit to the data using eqs S11–S14. Panel (f) shows the relationship between **λ** and CD strength (purple symbol) and a corresponding fit to the data (gray line). The error bars represent standard deviation from the fitting procedure.

The dependence of the FMR line width on the external magnetic field strength, which is reported in the plot as the resonance frequency *f*, at three different angles of the applied magnetic field relative to the sample normal are shown in Figure 2d for the NiFe/l-cys CdSe and in Figure 2e for the NiFe/d-cys CdSe. The line widths of the resonances, μ_0_Δ*H*, were obtained by fitting the spectra with a Lorentzian and asymmetric Lorentzian form (see eq S10 in the Supporting Information). When the field angle θ_*H*_ is 90°, the Δ*H* vs *f* plot is linear, however the Δ*H* vs *f* plot becomes dramatically nonlinear as θ_*H*_ decreases and approaches the normal to the film plane. In contrast, Δ*H* vs *f* remains linear and independent of the field angle θ_*H*_ for the achiral control NiFe/MPA CdSe (Figure 2c). The nonlinearity of the FMR line width versus field strength plot at a specific field angle θ_*H*_ arises from an increase of the damping factor α with angle. The angle dependence can be described by^35^

2 $$\alpha =\alpha \left(90^{{}^{\circ}}\right)+\lambda \;\cos^{2}\theta_{M}$$

where α(90°) is the damping factor for the external field perpendicular to the surface normal, λ is the dissymmetry factor for the chirality-induced anisotropic spin absorption, and θ*_M_* is the angle of the permalloy’s magnetization vector with respect to the surface normal. The difference between θ*_M_* and θ_*H*_, i.e., the spin polarization angle with respect to the surface normal, increases at lower magnetic fields (corresponding to lower frequencies) when the θ_*H*_ is tilted out-of-plane due to the demagnetization field of NiFe. As the magnitude of *H* increases at a fixed θ_*H*_, θ_*M*_ tends toward θ_*H*_. Note that the fits to these data include a parameter μ_0_Δ*H*_0_ to account for the inhomogeneous line width broadening caused by sample roughness and defects (see the Supporting Information for details). The solid lines in Figure 2c–e show a global fit to representative CdSe QD films and give λ values of 0.055 ± 0.013 for NiFe/l-cys CdSe, 0.038 ± 0.006 for NiFe/d-cys CdSe, and 0.002 ± 0.002 for the achiral control NiFe/MPA CdSe. For the NiFe/MPA, λ is essentially zero. The large difference in λ for the chiral and achiral CdSe QDs implies that the origin of the anisotropic damping factor arises from the chiral symmetry of the QDs which comprise the film. In addition to the difference in spin current absorption between chiral and achiral QDs, we find that reversing the spin polarization, i.e., changing *H* from a positive to a negative field, does not affect the spin current absorption in the chiral layer. The line width for both the NiFe/l-cys and NiFe/d-cys CdSe films overlap at positive and negative fields (see Supporting Information, Figure S5), i.e., both λ and α(90°) remain unchanged.

Next, we examined the correlation between the CD strength of the CdSe QDs and the corresponding chirality-induced anisotropic spin absorption factor λ of different CdSe QD films. Figure 2f plots the λ values versus the CD strength, measured as Δε of the first excitonic transition. The plots show a systematic variation of λ with CD strength; however, a V-shaped relationship manifests here, rather than the sigmoidal dependence found for the spin-polarized charge current. That is, the chiral CdSe QD films exhibit an increase in λ with an increase in the CD transition strength, |Δε|, but it does not depend on the sign of Δε. This behavior is consistent with that reported earlier for chiral cobalt oxide films.^35^ This work demonstrates that the anisotropic spin current absorption in the chiral CdSe QD film can be manipulated by changing the chiral strength of the CdSe QDs exciton transition.

## Discussion

The experiments described above show that both the spin-polarized charge transport, measured via mcAFM, and the pure spin current transport, measured via spin pumping, strongly correlate with the CD strength of the first exciton transition in the chiral QDs. For spin-polarized charge currents, the plot of %MR versus CD strength is described by a sigmoidal shape and its sign depends on the handedness of the chiral system, i.e., it is enantiospecific. In contrast, the pure spin current absorbed by a chiral film, as parametrized by the dissymmetry absorption coefficient λ, scales linearly with the CD strength and does not depend on handedness. Even when an external magnetic field reverses the sign of the pure spin current, the scattering strength remains unchanged, meaning that spin scattering between parallel and antiparallel spin polarization is not selective to the handedness. Although the two transport properties, pure spin current versus spin-polarized charge current, appear inconsistent on a superficial level, they can both be described by a universal chirality-induced spin splitting model as discussed below.

First, let us consider the case of pure spin current transport in the absence of net charge flow (Figure 3a). The spins injected from the permalloy film into the chiral layer dissipate through spin scattering or spin flip processes, and these dissipation processes are equivalent for opposite signs of spin polarizations, i.e., they are symmetric. These observations can be interpreted using a chirality-induced unconventional spin–orbit coupling (SOC) model (see refs 41 and 47). Figure 3a shows the energy versus momentum relation in the effective mass approximation with parabolic dispersion for spin splitting sub-bands in the case where the chirality leads to a SOC term that causes the zeroth order spin sub-bands to split and their energy minima to occur at a nonzero momentum. The displacement of the minimum along *k*_*z*_ and the energy stabilization depends on the magnitude of the structural chirality, e.g., the helicity parameter in the model from ref 47. Thus, the dependence of the energy shift and the splitting on the helicity of the material provides a rationale for the spin absorption coefficient λ being proportional to the CD strength. In addition, this mechanism predicts that no dependence of the selectivity on the sign of the spin, in agreement with the current experiment. The equivalent spin current absorption of both spin-up and spin-down states can be understood from considerations of time-reversal symmetry.^35^

**Figure 3:**
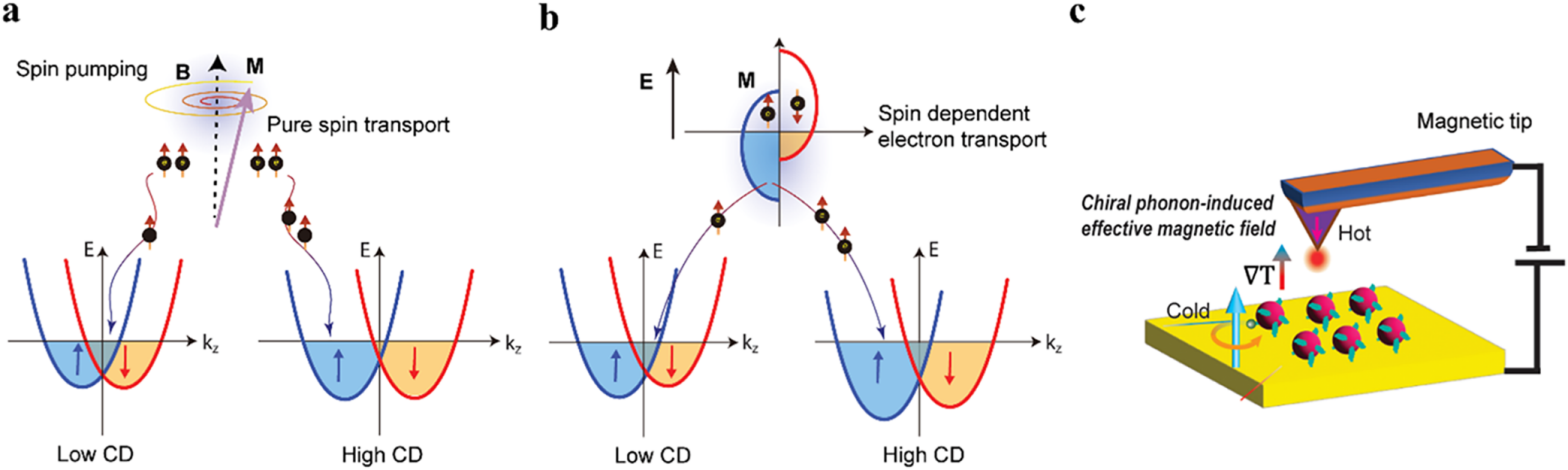
Chirality-dependent band splitting in transport measurements. Panel (a) shows dispersion curves, energy *E* versus wavevector *k*_*z*_, for the pure spin current injection case where a chiral structure causes the spin sub-bands to split because of chirality-induced unconventional spin–orbit coupling. As chirality is increased, e.g., low CD to high CD, the energy stabilizes and the displacement along ***k***_***z***_ increases. Panel (b) shows the splitting of spin sub-bands owing to chirality and their asymmetric stabilization due to an effective magnetic field arising from the charge current. In going from low CD to high CD, the difference in energy of the bands increases. Note that the spins (represented by the brown gradient arrow) and *k*_*z*_ are parallel to each other. Panel (c) shows a schematic of the temperature gradient generation in the mcAFM setup (indicated by the small arrow with gradient), causing the generation of a phonon-induced effective magnetic field.

In addition, the spin pumping experiments show that the spin injection depends on the direction of the applied magnetic field, with more efficient spin injection when the spin polarization is aligned with the film’s surface normal. This pronounced angle dependent selectivity is ∼7-fold for the chiral QD films in this work, whereas it was found to be ∼30-fold for the chiral cobalt oxide films.^35^ Such a strong dependence on the angle likely reflects the strength of chirality along the out-of-plane direction versus the in-plane direction of the films.

Now let us consider the injection of spin-polarized charge currents into a chiral system and its handedness-dependent MR responses (Figure 3b). In a simplified picture, the application of charge current in a chiral system leads to an energy shift in the spin-split bands. This shift generates net spin polarization, and this aspect of the CISS effect is like an unconventional collinear Edelstein effect. The appearance of an unchanging %MR polarity between positive and negative bias conditions (see Figure 1d), however, stands in stark contrast to that found for the Edelstein effect. That is, reversing the polarity of voltage bias in the mcAFM experiment inverts the charge current flow, which would produce an opposite spin polarization, and *MR* response if the Edelstein effect was manifesting. However, all of the mcAFM experiments, display the same polarity of the MR response across the bias range. These experimental facts suggest that the charge current must provide the rationale for breaking time-reversal symmetry in the chiral layer, but this symmetry breaking is independent of the current’s flow direction or the voltage bias.

How might this conundrum be resolved? Here, we propose that charge current flows through the chiral layer and generates a temperature gradient across the chiral-QD/AFM tip interface, arising from the mismatch in thermal conductivity between the two materials (Figure 3c). This subtle but necessary temperature gradient can excite chiral vibrational modes with a net angular momentum, resulting in the generation of an effective magnetic field with an orientation that depends on the handedness of the chiral material.^48^ Thus, the charge current gives rise to an effective magnetic field and further stabilizes one of the spin sub-bands. Even if the direction of the charge flow is reversed upon opposite bias, the temperature gradient across the interface remains unchanged, and therefore also the sign of the effective magnetic field from chiral vibration modes. Thus, the spin preference of the material relative to the momentum is fixed. The magnitude of the sub-band splitting is determined by the magnetization generated from the chiral helical field (unconventional spin–orbit coupling) of the CdSe QD film, which can be directly correlated to the CD strength. This scenario breaks time-reversal symmetry and gives rise to the handedness dependence of the spin-polarized charge current transport in mcAFM measurements (Figure 3b).

The explanation above uses the unconventional spin–orbit coupling (SOC) model together with the needed time-reversal symmetry breaking temperature gradient to rationalize the experimental observations in the mcAFM and FMR data. That is, we consider the spin selectivity to arise from a 2-fold mechanism that is a splitting of the sub-bands along the particle momentum direction and an enantiospecific stabilization of one of the spin sub-bands over the other because of the effective magnetic field generated by the temperature gradient associated with the application of charge current. These findings suggest that the correlation between the spin selectivity and the CD strength may arise from their mutual dependence on a SOC.

## Conclusions

This work demonstrates the correlation between CD strength and spin transport. The experiments show that the selectivity of the pure spin transport and spin-polarized charge transport increase with the CD strength, |Δε|, of the chiral QDs lowest energy exciton band, substantiating the use of CD as a descriptor for chiral-induced spin selectivity of transport properties. The spin-dependent charge current follows a sigmoid shape with Δε for its magnitude and is enantiospecific, i.e., its sign depends on the enantiomorph of the material. The pure spin current increases linearly with |Δε| and arises from the chirality, or helicity, along the transport direction, but is not enantiospecific; i.e., no sign dependence. These experimental observations can be rationalized by a chirality-induced unconventional spin–orbit coupling model and a temperature gradient generated by the charge current, offering a new perspective on the role of chiral symmetry and the electric field associated with the charge motion on the CISS effect. These findings deepen our understanding of the interplay between structural chirality and spin and offer insights that may be useful for design strategies with chiral materials in room temperature CISS spintronic applications.

## Methods

### Preparation of CdSe QDs

The synthesis of CdSe QDs was performed following a previously reported protocol.^42^ Briefly, 0.0514 g of CdO (Sigma-Aldrich) was mixed with 3.77 g of high-grade trioctylphosphine oxide (TOPO, Sigma-Aldrich) and 0.2687 g of octadecyl phosphonic acid (ODPA, Seqens) and was degassed under argon for 1h at 110 °C in a 50 mL 3-neck round-bottom flask. The reaction vessel was closed, and the temperature was then raised slowly to 300 °C under argon. When the color of the reaction mixture turned clear, the temperature was lowered to 270 °C, and a mixture of 0.316 g of Se (Sigma-Aldrich) dissolved in 3 mL of trioctylphosphine (Sigma-Aldrich) was injected. Aliquots from the reaction mixture were taken at regular intervals, and the peak wavelength from the UV–vis was used to monitor the growth of the QDs. Once the desired size was reached, the reaction was quenched by rapidly cooling. To purify the QDs, the reaction mixture was first centrifuged to remove unreacted materials, and then methanol was added to the supernatant to precipitate the QDs out of the solution. The QDs were centrifuged into a pellet and then redissolved in chloroform. Finally, the dispersed QDs were passed through a hydrophobic syringe filter and stored in the dark.

The purified ODPA-QDs were ligand exchanged according to a previously reported procedure.^31^ Briefly, the pH of a 3 mL aqueous solution of 0.74 mmol cysteine (Sigma-Aldrich) was adjusted to 11 using a concentrated tetramethylammonium hydroxide (Sigma-Aldrich) solution. Then, 3 mL of a 0.74 μM ODPA-capped CdSe QD solution was added to the cysteine solution, degassed, and stirred overnight. The aqueous phase was collected, and the ligand exchange QDs were purified through syringe and centrifugal filtration. For studies at variable CD strengths of QDs, this was accomplished by varying the cysteine concentration between 0.74 and 0.14 mmol, and by varying the enantiopurity between 100% and 20%. Synthesis of achiral CdSe followed a similar procedure; however, mercaptopropionic acid instead of cysteine was used during the ligand exchange.

### Formation of CdSe Films for mcAFM

An amine-terminated SAM was formed by incubating a (50 nm) gold-coated silica substrate in a 5 mM solution of 8-amino-1-octanethiol in ethanol for 24 h. Ellipsometry measurements at 5 different locations along the substrate were used to estimate a thickness of ∼ 0.9 nm height (see Table S1), in good agreement with the expected height for SAMs formed with molecules at a tilt angle of 30°.^49^ 100 μL of a 5 nM CdSe QD solution was dropcast onto the SAM coated surface and dried prior to AFM measurements. A nanoshaving analysis was performed to confirm the monolayer formation as follows: (1) the AFM tip was scanned in contact mode with a force of approximately 250 nN to remove the assembly from the scanned area (*ca*. 500 nm^2^) and (2) an AC mode scan on a larger area, centered around the same region in step 1 was performed. Figure S2a shows a representative topography image from step 2 in which the dark square corresponds to the nanoshaved region of the thin film. Figure S2b shows a corresponding height profile recorded for the highlighted area in the figure. The difference in height between the film surface (area between 0–2 μm) and the nanoshaved region (area between 2.2–2.6 μm) is ∼3 nm, consistent with the size of the (∼2.3 nm) and thickness of the SAM (∼0.9 nm).

### mcAFM Measurements

The mcAFM measurements were performed on an Asylum MFP3D instrument using a Co/Cr tip with a force constant of 2.8 N/m. Experiments comprised a biased nonmagnetic substrate and a ferromagnetic tip. Three sets of curves were collected for each QD assembly by alternating the magnetization of the tip, *e.g*., North, South, followed by North again. For each magnetization the average *i*-*V* response from 30 different locations was acquired and a data set is only considered ‘reproducible’ if the first and the third set of curves, *i.e*., those done with the same magnetization, are within the 95% confidence intervals. This criterion was met ∼70% of the time.

### Formation of Thin Films for FMR

The thin films for FMR measurements were prepared on a 1 cm x 1 cm glass substrate by spin-coating dilute solutions of chiral CdSe in methanol at a speed of 3000 rpm for 30 s. The thickness of the film was measured using profilometry, and the concentration of the stock solution was adjusted to prepare a film of the desired thickness.

## Supplementary Material



## Notes

Optical Characterizations of QDs, Ellipsometry analysis of 8-amino-1-octanethiol SAM, Characterizations of QD film by AFM, Mechanism for mc-AFM measurements, Calculations for extrapolating CD v/s MR plot, FMR measurements, FMR at varying magnetic fields (PDF)
