# Supplementary material for: Interplay between Chiro-Optical and Spin Transport Properties in Chiral CdSe Quantum Dots
Source: ACS Nano. 2025 Sep 22;19(39):35119–26. doi: 10.1021/acsnano.5c12765 (PMC12509305; doi:10.1021/acsnano.5c12765)
Supplement: Supplementary file 1 [file nn5c12765_si_001.pdf]

Supplementary information for:

Interplay Between Chiro-optical and Spin Transport Properties in Chiral CdSe Quantum Dots

Elizabeth Shiby,[1*] Rui Sun[2*], Brian P. Bloom,[1] Joseph A. Albro,[1] Dali Sun,[2†] David H. Waldeck[1†]

[1] Department of Chemistry, University of Pittsburgh, Pittsburgh, Pennsylvania 15260, United States
[2] Department of Physics, North Carolina State University, Raleigh, North Carolina 27695, United States

[*]These authors contributed equally to this work

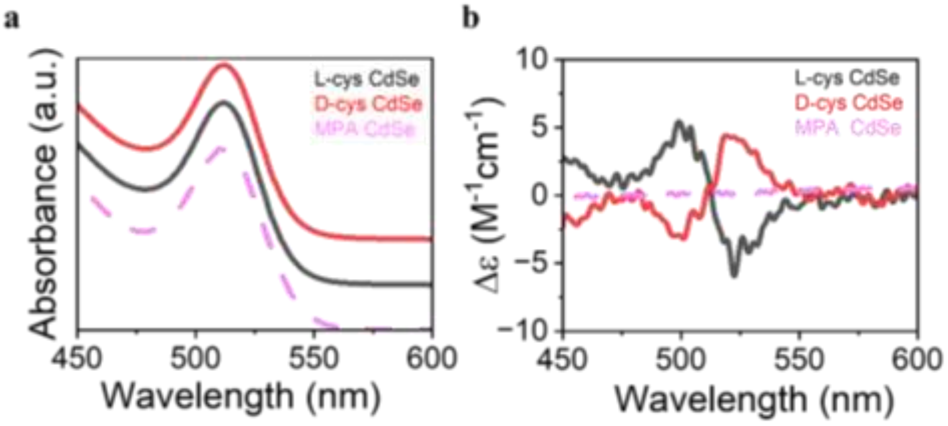

Figure S1. Optical Characterizations of QDs. Absorbance (**a**) and circular dichroism spectra (**b**) of L-cys CdSe (black), D-cys CdSe (red), and MPA CdSe (pink) QDS. The absorbance data is offset for clarity.

Table S1: Ellipsometry analysis of 8-amino-1-octanethiol SAM

Trial	SAM height(nm)
1	1.19
2	0.88
3	0.78
4	1.18
5	0.49
Average	0.91± 0.26

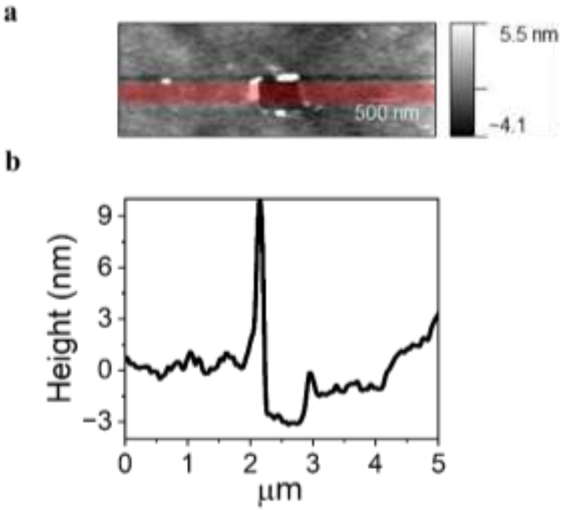

Figure S2. Characterizations of QD film by AFM. Panel (**a**) shows an AFM topography image of a representative CdSe QD-SAM assembly following nanoshaving. Panel (**b**) shows the average height profile of the thin film across the nanoshaved area.

S1: Mechanism for mc-AFM measurements

For the spin-polarized charge current measurements using mcAFM, a lower resistance is observed for one magnetization and a higher resistance for the opposite magnetization. The change in current response is determined by the spin selectivity of the chiral material and the direction of the charge current, i.e. from the tip to the substrate or from the substrate to the tip. Figure S3b shows a cartoon for this process using a chiral material that preferentially transports spins antiparallel to their momentum of the electron. Upon magnetization of the tip, the spin sub bands split such that one spin sub band becomes majority filled and the other majority empty. Which spin sub band is more or less filled depends on the orientation of the applied external magnetic field (North vs. South). In the diagram below this is represented by the yellow and blue areas under the Fermi level. For experiments operating under negative bias conditions, electrons flow from the substrate, through the chiral material, and to the tip. Note that the spin preference of the chiral material is indicated by a yellow arrow. When the magnetization of the tip is such that the majority of unoccupied spins (blue) is opposite to that of the chiral material's spin preference for transport (yellow) a higher resistance / lower current is observed; See Figure S3b top left. Conversely under the opposite magnetization (figure S3b top right), the majority of unoccupied spin sub band states is yellow and therefore a higher current is observed. Under positive bias conditions, electrons transfer from the tip, through the chiral material, and into the substrate. Because the momentum of the electron is now opposite, the opposite spin preference of the chiral material becomes manifest; i.e. a blue arrow is above the material in the diagram. For a tip magnetization in which the spin sub-bands are majority yellow populated (Figure S3b, bottom left), a higher resistance is observed, whereas when the tip is magnetized such that the spin sub-bands are majority blue occupied (Figure S3b, bottom right), a higher current is observed.

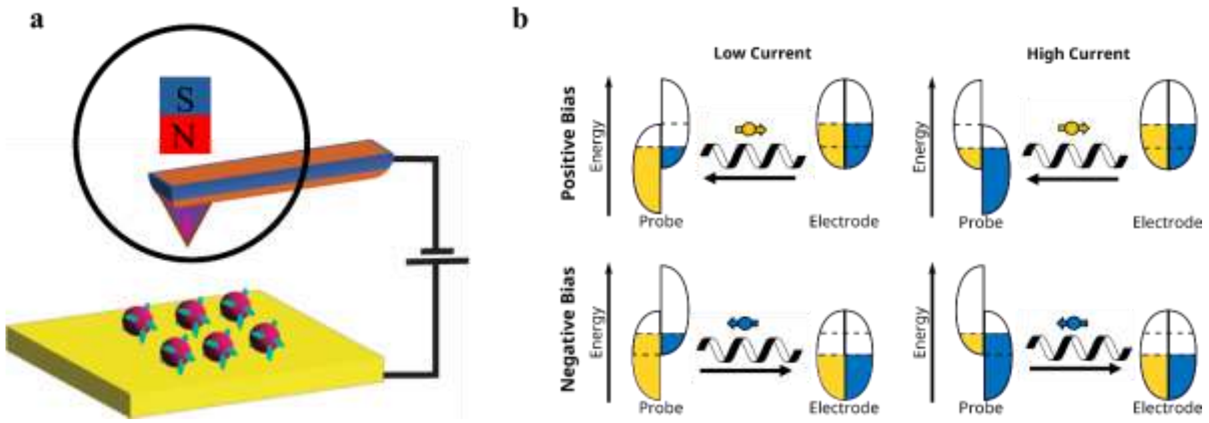

Figure S3. mcAFM mechanism. Cartoon demonstrating (**a**) the magnetization direction of the tip when magnetized with the north pole of a permanent magnet and (**b**) the working mechanism of a CISS measurement using mcAFM for a chiral material that preferentially transmits spins antiparallel to their momentum (depicted by blue or yellow arrows on the top). Note that the solid black arrows show the direction of electron velocity.

S2: Calculations for extrapolating CD v/s MR plot

Because the AFM tip ($ca.$ 84 nm) is much larger than that of an individual QD ($ca.$ 2.5 nm), the negative space between QDs in an assembly will artificially reduce the magnetoresistance in our measurements. Figure S3a shows the %MR determined through mc-AFM without the correction factor. To account for the void space between QDs, we systematically varied the mole fraction of chiral and achiral particles in the assembly and measured the resulting change in magnetoresistance using mcAFM. Figure S3b shows a linear relationship with the fraction of chiral: achiral QDs.

To model this behavior, consider N_c and N_a as the number of chiral and achiral QD with radius r in a close-packed square arrangement. If we treat each QD as a sphere, then the area occupied by the chiral and achiral particles will be $(N_c + N_a)\pi r^2$and the area occupied by the vacancies can be written as $(4-\pi)r^2(N_a + N_c)$. The magnetoresistance from the QD assembly, including chiral and achiral particles with vacancies, is then expressed as the ratio of the difference in current between the South and the North magnetizations of the tip to the total current such that

$$\% MR_{exp} = \frac{N_c \,\pi r^2(i_S - i_N)}{N_c \,\pi r^2(i_S + i_N) + N_a \,\pi r^2(i_{a,S} + i_{a,N}) + (4-\pi) r^2 (N_a + N_c)(i_{vac,S} + i_{vac,N})} \qquad (S1)$$

where i_S and i_N are the current passing through the chiral QDs with South and North magnetization, $i_{a,N}$ and $i_{a,S}$ are the currents passing through the achiral with South and North magnetization, and $i_{vac,S}$ and $i_{vac,N}$ are currents passing through the vacancies with North and South magnetizations, respectively.

Transforming the number of chiral and achiral molecules into mole fractions, X_a and X_c, is accomplished by defining

$$X_a = \frac{N_a}{N_a + N_c} \quad \text{and} \quad X_c = \frac{N_c}{N_a + N_c}$$

Using this substitution transforms equation ($S1$) into the form

$$\% MR_{exp} = \frac{X_c \,\pi (i_S - i_N)}{X_c \,\pi (i_S + i_N) + X_a \,\pi (i_{a,S} + i_{a,N}) + (4-\pi)(i_{vac,S} + i_{vac,N})} \qquad (S2)$$

Rearranging and combining common terms in the equation yields

$$\%MR_{exp} = \frac{X_c\,(i_S - i_N)}{X_c\,(i_S + i_N) + X_a\,(i_{a,S} + i_{a,N}) + \left(\frac{4}{\pi} - 1\right)(i_{vac,S} + i_{vac,N})} \qquad (S3)$$

Because $X_c = 1 - X_a$ and $X_a = 1 - X_c$ then ($S3$) can be rewritten as

$$\%MR_{exp} = \frac{X_c\,(i_S - i_N)}{X_c\,(i_S + i_N) + (i_{a,S} + i_{a,N}) - X_c\,(i_{a,S} + i_{a,N}) + \left(\frac{4}{\pi} - 1\right)(i_{vac,S} + i_{vac,N})} \qquad (S4)$$

Dividing ($S4$) by the sum of the current response associated with transport through the chiral QDs, $(i_S + i_N)$, gives

$$\%MR_{exp} = \frac{X_c\,MR_{true}}{X_c + \frac{(i_{a,S} + i_{a,N})}{(i_S + i_N)} - X_c\frac{(i_{a,S} + i_{a,N})}{(i_S + i_N)} + \left(\frac{4}{\pi} - 1\right)\frac{(i_{vac,S} + i_{vac,N})}{(i_S + i_N)}} \qquad (S5)$$

where $\%\,MR_{true} = \frac{(i_S - i_N)}{(i_S + i_N)}$. If $(i_S + i_N) = (i_{a,S} + i_{a,N})$, then

$$\%MR_{exp} = \frac{X_c\,\%MR_{true}}{1 + \left(\frac{4}{\pi} - 1\right)\frac{(i_{vac,S} + i_{vac,N})}{(i_S + i_N)}} \qquad (S6)$$

Let us define $\frac{(i_{vac,S} + i_{vac,N})}{(i_S + i_N)} = R$. When $R \rightarrow 0$ equation ($S6$) becomes,

$$\%MR_{exp} = X_c\,\%MR_{true} \qquad (S7)$$

which corresponds to the ideal case that has no vacancies. When $R \rightarrow 1$

$$MR_{exp} = X_c\,\frac{\pi}{4}\,\%MR_{true}\ (S8) \qquad \text{or} \qquad \%\frac{MR_{true}}{MR_{exp}} = X_c\,\frac{4}{\pi} \qquad (S9)$$

From the slope in Figure S4b and the above equations, the experimental and calculated values differ by a factor of 1.3 for $X_c = 1$. Therefore, the magnetoresistance in Figure S4a, measured at Xc = 1, is corrected to account for vacancies between the QDs by multiplying by 1.3 and is replotted in Figure 1d.

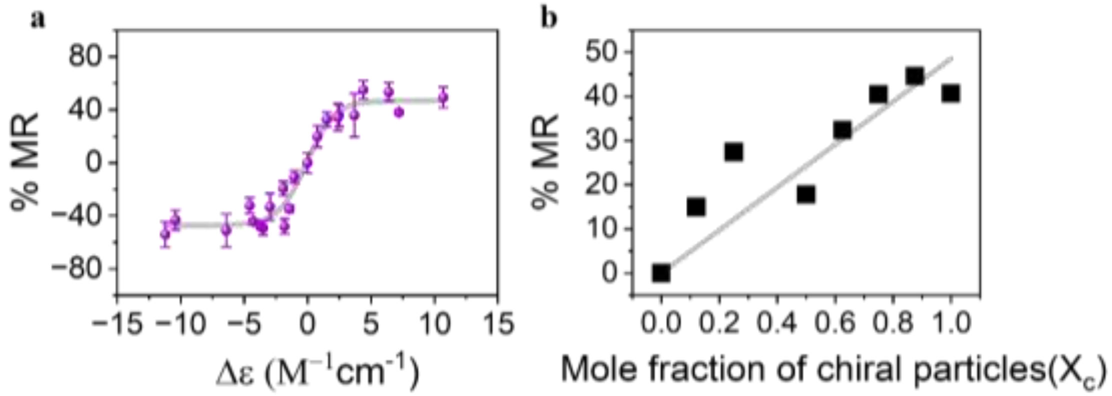

Figure S4. Contribution of vacancies to %MR. Panel (**a**) plots the as-measured magnetoresistance from mc-AFM of chiral CdSe QDs without the correction term versus the molar circular dichroism. Panel (**b**) plots the mole fraction of chiral particles in a film to the mc-AFM measured magnetoresistance.

S3: FMR measurements

The FMR measurements were conducted using a custom-built setup with an Anritsu VNA generating the microwave signal. The microwave absorption spectra as a function of the external magnetic field were obtained by monitoring the transmission parameter S_{21}, which is a combination of symmetric and antisymmetric Lorentzian components described by the equation:

$$Re\,\Delta S_{21}\,(H) = a_1 + b_1 * \frac{(\Delta H)^2}{(H-H_{\text{FMR}})^2+(\Delta H)^2} + b_2 * \frac{\Delta H*(H-H_{\text{FMR}})}{(H-H_{\text{FMR}})^2+(\Delta H)^2} \qquad (S10)$$

where Re ΔS_{21} is the real part of transmission parameters, ΔH represents the linewidth, H_{FMR} denotes the resonance field and a_i , b_i are coefficients. From this equation, the resonance field H_{FMR} and linewidth at specific frequency can be extracted.

For a magnetic system, the total magnetic free energy density can be expressed as

$$F = -\mu_0 H M_s[\sin\theta_H\ \sin\theta\ \cos(\varphi-\varphi_H)\ +\cos\theta_H\cos\theta]$$
$$-\mu_0 M_s^2\sin^2\theta + K_{in}\sin^2\theta + K_u\sin^2\theta\ \sin^2(\varphi-\varphi_0) \qquad (S11)$$

where K_u and K_{in} are the in-plane two-fold anisotropy and interfacial perpendicular anisotropy constants, respectively. The magnetization direction, M_s, is determined by an angle, θ, with respect to the z-axis and by an angle, φ, between the direction of the M_s vector projection on the x-y plane and the y-axis.

The relationship between H_{FMR} and frequency f is given by

$$f = \frac{\gamma}{2\pi M_s\sin\theta}\sqrt{F_{\theta\theta}F_{\varphi\varphi} - F_{\theta\varphi}^2} \qquad (S12)$$

where $F_{\theta\theta} = \frac{\partial^2 F}{\partial\theta^2}$, $F_{\varphi\varphi} = \frac{\partial^2 F}{\partial\varphi^2}$, and $F_{\theta\varphi} = \frac{\partial^2 F}{\partial\theta\partial\varphi}$. By combining equations ($S11$) and ($S12$), the linewidth ΔH as a function of frequency f and the frequency f as a function of resonance field H_{FMR} can be globally fitted, as illustrated in Figures S5c and S5d.

The relationship between $\mu_0\Delta H$ and f can be described by [1]:

$$\mu_0\Delta H = \frac{\alpha\gamma(F_{\theta\theta}+\frac{1}{\sin^2\theta}F_{\varphi\varphi})}{M_s\,|d\omega/dH|} + \mu_0\Delta H_0 \qquad (S13)$$

where γ is the gyromagnetic ratio of NiFe, ω is the resonance frequency ($\omega = 2\pi f$) and $\mu_0\Delta H_0$ is the inhomogeneous broadening linewidth caused by sample roughness, defects, or orientation of crystallites. For a magnetic system without strong magnetic anisotropy and anisotropic damping factor, the damping factor α is invariant. In the chiral system, the damping factor α exhibits an anisotropic behavior, which is described as

$$\alpha = \alpha' + \lambda\cos^2\theta_M. \qquad (S14)$$

Here, the α' is the damping factor measured with the magnetic field perpendicular to the surface normal. It represents the damping factor of NiFe itself and additional damping factor from spin pumping effect induced by the conventional SOC effect.

As shown in Figure S6, we also compared the linewidth ΔH vs. f at positive and negative field at different field angles θ_H. No matter if the magnetic field is in-plane or tilted to the surface normal direction, the

linewidth ΔH at positive and negative fields are exactly overlapped with each other at different frequency points. It demonstrates that the spin current injection and absorption does not rely on the spin polarization direction in the presence of structural chirality.

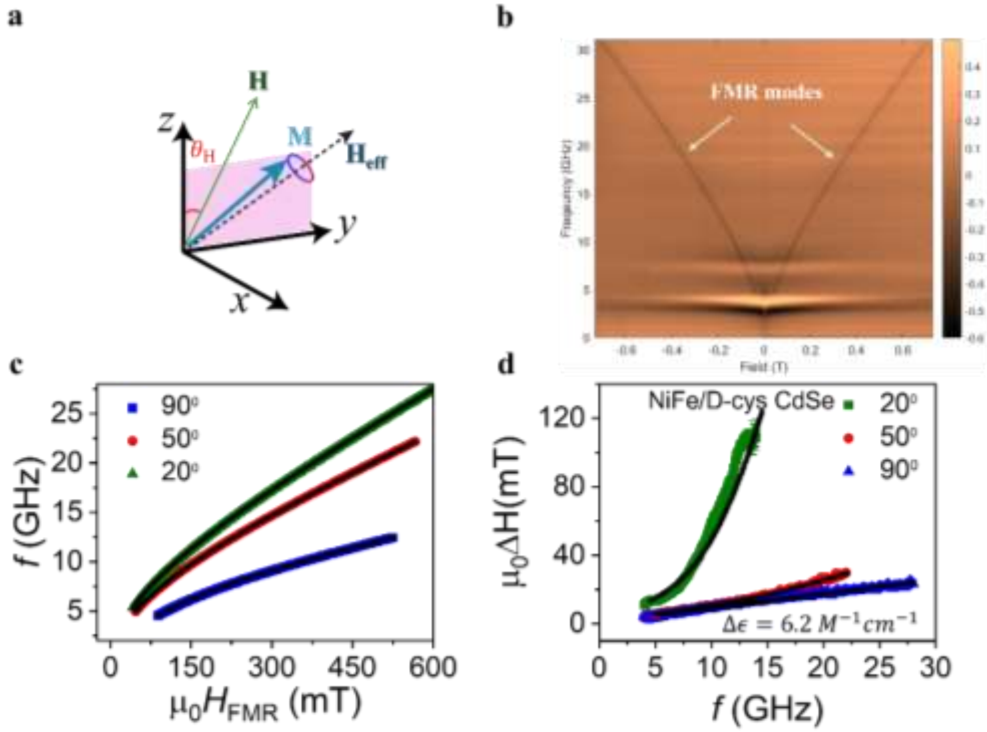

Figure S5. FMR measurements. (**a**) Coordinate system used in VNA-FMR. (**b**) The 2D plot of VNA-FMR spectra for the NiFe/D-cys CdSe measured at θ_H at 90°. (**c**) & (**d**) f vs. resonance field H_{FMR} and the obtained linewidth ΔH as a function of f at different θ_H, respectively. The solid lines are global fits to the data using Equations *S12-S14*.

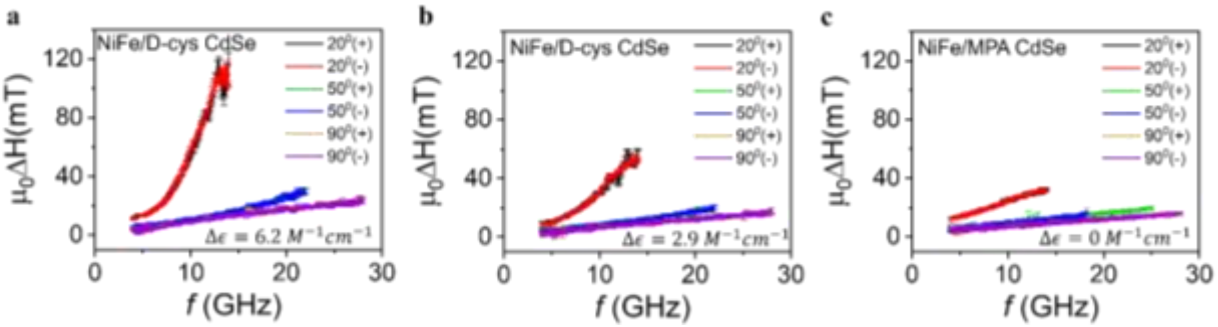

Figure S6. FMR at varying magnetic fields. The obtained linewidth ΔH as a function of f at different θ_H for NiFe/D-cys CdSe samples with varying CD strength (Panels **a** & **b**) and for achiral NiFe/MPA CdSe (Panel **c**). The results in the positive and negative fields are identical for the chiral and achiral samples.

References

(1) Suhl, H. Ferromagnetic Resonance in Nickel Ferrite between One and Two Kilomegacycles. *Phys. Rev.* **1955**, *97*, 555.
